# Advances in the Study of the Pathology and Treatment of Alzheimer’s Disease and Its Association with Periodontitis

**DOI:** 10.3390/life13112203

**Published:** 2023-11-13

**Authors:** Dan Tang, Chang Sun, Jumei Yang, Lili Fan, Yonggang Wang

**Affiliations:** 1 School of Life Science and Engineering, Lanzhou University of Technology, Lanzhou 730050, China; sc20000214@163.com (C.S.); fanll1779380@163.com (L.F.); 2 Lanzhou University Second Hospital, Lanzhou 730000, China; 18893472337@163.com

**Keywords:** Alzheimer’s disease, pathogenesis, treatment, periodontitis, *Porphyromonas gingivalis*

## Abstract

Alzheimer’s disease (AD) has become one of the leading causes of health problems in the elderly, and studying its causes and treatments remains a serious challenge for researchers worldwide. The two main pathological features of Alzheimer’s disease are the extracellular deposition of β-amyloid (Aβ) to form senile plaques and the intracellular aggregation of hyperphosphorylated Tau protein to form neurofibrillary tangles (NFTs). Researchers have proposed several hypotheses to elucidate the pathogenesis of AD, but due to the complexity of the pathophysiologic factors involved in the development of AD, no effective drugs have been found to stop the progression of the disease. Currently, the mainstay drugs used to treat AD can only alleviate the patient’s symptoms and do not have a therapeutic effect. As researchers explore interactions among diseases, much evidence suggests that there is a close link between periodontitis and AD, and that periodontal pathogenic bacteria can exacerbate Aβ deposition and Tau protein hyperphosphorylation through neuroinflammatory mechanisms, thereby advancing the pathogenesis of AD. This article reviews recent advances in the pathogenesis of AD, available therapeutic agents, the relevance of periodontitis to AD, and mechanisms of action.

## 1. Introduction

In 2021, the world entered a population aging stage, and with the new longer life expectancy, dementia has become a major health issue for older adults worldwide. According to the World Alzheimer’s Disease Report 2021, more than 55 million people worldwide currently have dementia [1], and this number is expected to increase to 152 million by 2050 [2]. About 70% of dementia is caused by Alzheimer’s disease [3]. AD is the most common form of dementia and is a complex and irreversible neurodegenerative disease. Its main manifestation is the progression of patients from mild cognitive impairment (MCI) to severe mental impairment as they age [4]. AD is divided into early-onset AD (EOAD) and late-onset AD (LOAD). EOAD is a very uncommon condition associated with genetic factors such as mutations in susceptibility genes like amyloid precursor protein (APP), apolipoprotein E4 (ApoE4), presenilin 1 (PSEN1), and presenilin 2 (PSEN2), which can lead to an overproduction of amyloid beta. LOAD (more than 90%) is associated with environmental and risk factors such as age, gender, lifestyle, education, diet, substance abuse, disease history, and family history [5].

Since 1906, when German neuropathologist Dr. Alzheimer reported the first case of AD, various hypotheses have been developed from pathophysiology and other aspects, mainly including cholinergic hypothesis, β-amyloid cascade hypothesis, Tau protein phosphorylation hypothesis, neuroinflammation hypothesis, etc. [6]. However, due to the complexity of the pathophysiological factors involved in the development of AD, and with various pathological changes occurring in the years preceding—and in the decade following—cognitive decline [7], the exact pathogenesis has not yet been elucidated, and available treatments and clinical drugs are scarce. Therefore, the study of AD pathogenesis and potential causative factors will be of great benefit to the search for new therapeutic pathways and strategies in the future and will be of great importance to the protection of the health of the elderly.

About a decade ago, S. Poole et al. [8] detected the presence of *Porphyromonas gingivalis* LPS in the brains of AD patients by studying the bacterial composition of periodontal disease in the brain tissue of AD patients 12 h after death. In a review by A.R. Kamer et al. [9], it was also suggested that periodontal-derived pro-inflammatory molecules and *Porphyromonas gingivalis* can enter the brain through the somatic circulation or neural pathways and increase Aβ synthesis, leading to Aβ deposition and cognitive dysfunction. N. Ishida et al. [10] found that cognitive function was impaired in mice infected with *Porphyromonas gingivalis* compared to control mice. All of these ideas suggest a correlation between periodontitis and the onset and progression of AD. Although the involvement of periodontitis in the pathogenesis of AD is not yet clear, it points to new directions for exploring therapeutic avenues for AD.

This paper summarizes the pathogenesis of AD and current principal therapeutic drugs and reviews the progress of research on the link between AD and periodontitis to provide ideas for the development of new ways to treat AD.

## 2. Pathogenesis of Alzheimer’s Disease

The two main pathological features of Alzheimer’s disease that are recognized worldwide are the formation of senile plaques via extracellular deposition of β-amyloid (Aβ) and neuronal fibrillary tangles (NFTs) via intracellular hyperphosphorylation and the aggregation of Tau protein [11]. In response to these identified pathological features, researchers have been investigating the pathogenesis of AD and have proposed various hypotheses, including the cholinergic hypothesis, the β-amyloid cascade hypothesis, the Tau protein phosphorylation hypothesis, and the neuroinflammatory hypothesis [12,13].

### 2.1. Cholinergic Injury Hypothesis

The cholinergic damage hypothesis was the first hypothesis proposed for the pathogenesis of AD, which suggests that memory and cognitive dysfunction in AD patients is mainly related to cholinergic neuronal damage and reduced acetylcholine (Ach) levels [12]. Cholinergic neurons in the normal basal forebrain synthesize large amounts of Ach and deliver it to the cerebral cortex and hippocampus via projection fibers. The hippocampus is the central region of the human brain associated with learning, memory and cognition, and is also the first area to be damaged in AD [14]. Ach is the main neurotransmitter controlling learning and memory functions in the hippocampus and has a role in facilitating neurotransmission and long-term potentiation (LTP) [15]. However, in AD patients with a significant decrease in cholinergic neurons in the basal forebrain, the synthesis and release of Ach and the number of nicotinic Ach receptors (nAChRs) are also reduced, ultimately leading to impaired learning and cognitive function [16].

### 2.2. The β-Amyloid Cascade Hypothesis

The β-amyloid (Aβ) cascade hypothesis originated in the 1980s and is one of the most widely known mechanisms for the pathogenesis of Alzheimer’s disease [17]. The Aβ cascade hypothesis proposes that the neurodegeneration of AD is caused by an abnormal deposition of β-amyloid plaques in various regions of the brain due to an overproduction of Aβ or failure of its clearance mechanisms [18,19,20,21,22]. Generally, in a young brain, Aβ production and elimination are kept in balance [23], and the Aβ produced is soluble. However, this equilibrium is disrupted by aging and neurodegeneration, resulting in Aβ forming insoluble proteins and depositing them in the brain [24,25,26,27,28], affecting neuronal transmission and impairing memory [29,30].

The amyloid precursor protein (APP) produces Aβ [31,32]. APP is a single channel receptor-like type I transmembrane glycoprotein that plays an important role in neural growth, migration, and maturation during different stages of brain development [33,34,35]. APP is located on human chromosome 21 and contains 18 exons, which are selectively spliced to produce three major isoforms: APP695, APP751, and APP770. APP695 is mainly expressed in the brain [36,37]. APP undergoes sequential protein hydrolytic cleavage during intracellular transport and is cleaved in both amyloid and non-amyloid pathways [38,39].

The non-amyloid pathway is the process by which APP is cleaved by α-secretase. When APP is catabolized by α-secretase at the cell surface, a soluble APP-α (sAPPα) fragment and a C-terminal fragment 83 (αCTF83) are produced. Then, αCTF83 is cleaved by γ-secretase to produce an extracellular P3 fragment and amino-terminal APP intracellular domain (AICD50) [37,40,41]. About 90% of APPs are cleaved by α-secretase [42].

**Figure 1:**
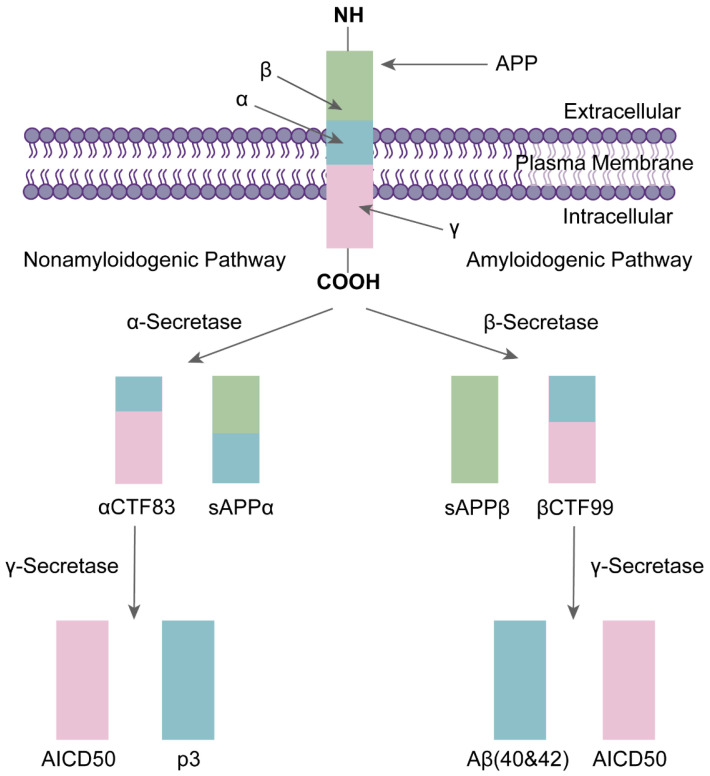
The formation of Aβ: APP cleavage by β-secretase produces soluble fragments sAPPβ and βCTF99, and βCTF99 cleavage by γ-secretase produces Aβ40/Aβ42 and amino-terminal APP intracellular domain (AICD50) [32].

The amyloid pathway refers to the cleavage of APP by two transmembrane enzymes, namely β-secretase and γ-secretase (a multimeric protein complex) [43]. γ-secretase consists of four subunits: Presenilin (PS), Presenilin enhancer 2 (PEN-2), APH-1, and Nicastrin (NCT) [44]. APP is cleaved by β-secretase within the lipid bilayer to produce a soluble APP-β (sAPPβ) and β C-terminal fragment 99 (βCTF99). Subsequently, βCTF99 is cleaved by γ-secretase to produce the Aβ and AICD50 (Figure 1). Since this process is not precise, the resulting Aβ varies in size [37,40,45]. Aβ polypeptides of 40 and 42 amino acids in length are the predominant forms [46], with Aβ40 accounting for 90% of the total, but Aβ42 is more aggregated and more neurotoxic than Aβ40 [47,48]. The generated Aβ forms neuroinflammatory plaques in the vasculature and parenchyma by self-aggregating to form oligomers of different sizes [49]. These oligomers and plaques are neurotoxic and can trigger various immune inflammatory responses and neurotoxic cascades, causing neuronal degeneration and even death. For example, they interfere with synaptic function and thus promote neuroinflammatory processes; or they increase Ca^2+^ levels in mitochondria, leading to abnormal mitochondrial function [30,50].

### 2.3. Mechanism of Tau Protein Hyperphosphorylation

The Tau protein is an important axonal microtubule-associated protein (MAP) that is widely distributed throughout the central nervous system (CNS) and accounts for approximately 80% of MAP [51,52,53]. In humans, the Tau protein is located on chromosome 17q21 and has six isoforms [54]. Tau proteins are highly soluble [55] and are essential for regulating and promoting proper microtubule assembly, nutrient transport, protein synthesis, neuroprotection, and apoptosis, and they play a key role in maintaining the stability of neuronal microtubules and regulating axonal growth and transport [56,57,58]. Tau proteins in the normal mature brain contain only two to three phosphate groups, but an abnormal phosphorylation process leads to the conversion of Tau proteins into hyperphosphorylated Tau proteins (P-Tau) (five to nine phosphate groups per molecule of P-Tau protein) and the loss of their normal biological functions. The P-Tau is misfolded and aggregated to form neuronal fiber tangles (NFT), which are one of the typical pathological features of AD [3,59].

**Figure 2:**
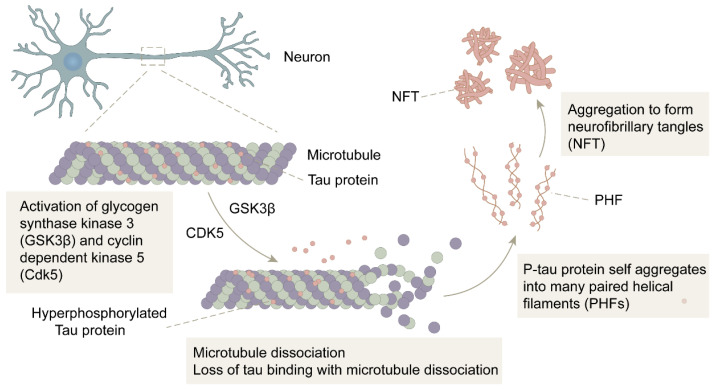
The formation of NFT: In the presence of GSK3β and CDK5, the Tau protein is hyperphosphorylated into P-Tau. After microtubule disassembly, the P-Tau loses its binding to microtubules and self-aggregates into multiple pairs of helical filaments (PHF), further forming neurofibrillary tangles (NFT).

There is a close link between Tau protein hyperphosphorylation and Aβ. David E.H. et al. [60] found that Aβ aggregation accelerated Tau protein hyperphosphorylation and NFT formation. In a study by Götz.J et al. [61], that Aβ was also clearly shown to be able to lead to the phosphorylation of Tau protein and promote the formation of NFT. All these studies demonstrate that Tau protein hyperphosphorylation is a downstream event of Aβ deposition [60,61,62]. In the normal brain, kinases that phosphorylate Tau proteins and dephosphorylated phosphatases together regulate the phosphorylation process of Tau proteins, and the activities of the two enzymes are in relative balance. However, in the brains of AD patients, the increased concentration of Aβ activates two major Tau kinases: glycogen synthase kinase 3β (GSK3β) and cyclin-dependent kinase 5 (CDK5), which promote the process of Tau protein phosphorylation. The imbalance of Tau kinase and phosphatase activities induces and accelerates the production of the P-Tau protein, which self-aggregates into many paired helical filaments (PHF) and further competes for binding to form NFTs, ultimately leading to microtubule destabilization and impaired neuronal transport (Figure 2). Moreover, the low affinity of the P-Tau protein for microtubules leads to both malfunctioning communication among neurons and apoptosis [6,63].

Researchers use age spots formed by Aβ deposits as a sign of early disease and NFT formed by P-Tau protein aggregation as a sign of late disease [64]. On the one hand, Aβ can cause the hyperphosphorylation of Tau proteins. With the increase in P-Tau protein content, the immune response to Aβ is also significantly higher. On the other hand, the Tau protein is necessary for the neurotoxicity of Aβ. Aβ can only cause a degeneration of Tau protein-containing neurons, and in the absence of the Tau protein, Aβ does not cause neurodegenerative mechanisms that lead to memory impairment [42]. Both synergistically advance disease progression in neurodegeneration and AD.

### 2.4. Neuroinflammatory Mechanisms

Neuroinflammation also plays a crucial role in the progression of neurodegeneration in AD. In the early stages of the disease, neuroinflammation only occurs as a self-defense response of the immune system to pathogens or stimuli. However, when this response turns into chronic neuroinflammation, it can increase the production of neurotoxic mediators, promote synaptic loss, result in axonal transport defects, and cause mitochondrial dysfunction, ultimately leading to neuronal death and cognitive dysfunction [65,66,67,68]. Cytokines secreted by activated microglia and astrocytes are major players in neuroinflammatory mechanisms [69].

Microglia are found in the CNS and account for approximately 10–15% of all glial cells, are highly specific innate immune cells, and are resident macrophages in the brain [70,71,72]. Microglia exert their protective effects on brain tissue by removing cellular debris and infection factors, and they also influence learning and memory functions by regulating synaptic strength, which is important for regulating synaptic plasticity, maintaining normal intracerebral environmental balance, and supporting brain development [73,74]. Under normal physiological conditions, unactivated microglia called M0-type microglia remain in a resting state. In an AD brain, deposited Aβ plaques can activate microglia. Activated microglia can be divided into a pro-inflammatory phenotype, M1, and an anti-inflammatory phenotype, M2. The M2 phenotype is dominant in the early stages of inflammation, but as the duration of inflammation increases, the proportion of the M1 phenotype gradually exceeds that of the M2 phenotype [75]. M1 phenotype microglia respond to damage caused by Aβ deposition by releasing pro-inflammatory factors, such as interleukins IL-1β, IL-6, IL-9, IL-10, tumor necrosis factor-α (TNF-α), and reactive oxygen species (ROS), which cause peripheral neuronal damage. Microglia in pathological conditions are over-activated, increasing the release of pro-inflammatory factors and leading to their excessive accumulation, which eventually develops into chronic neuroinflammation, and which in turn leads to neuronal death and drives the disease progression of AD [76,77,78,79,80,81,82]. Activated microglia can drive Tau protein hyperphosphorylation and NFT formation [83,84].

**Figure 3:**
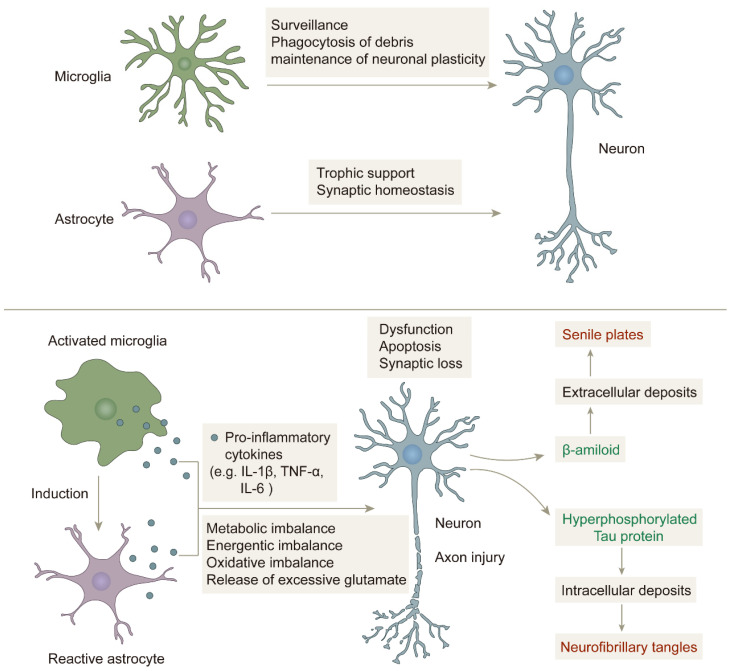
The role of microglia and astrocytes in neuroinflammation: in a non-inflammatory state, microglia and astrocytes play their respective roles and work together to protect neurons. In an inflammatory state, microglia are activated, and astrocytes are induced to become reactive astrocytes, which together release pro-inflammatory factors that cause imbalance in the brain environment and damage neurons, eventually forming senile plaques and neurofibrillary tangles [92].

Astrocytes are astral in shape and are the most numerous glial cell subtype in the central system [85]. Astrocytes are involved in a variety of physiological processes, including synaptogenesis and transmission, the regulation of synaptic plasticity, neurotransmitter delivery, the regulation of metabolism, and the maintenance of ionic homeostasis, and they are also involved in forming the blood–brain barrier (BBB) and maintaining its permeability [86] as well as providing nutritional support to neurons, which is essential for the functioning of the central system [87]. By contrast, in the case of inflammatory injury, astrocytes are induced by activated microglia and immediately undergo a process of astrocyte proliferation to reactive astrocytes. Reactive astrocytes have two phenotypes: the neurotoxic A1 phenotype and the protective A2 phenotype. Like microglia, reactive astrocytes release pro-inflammatory factors, chemokines, complement factors, and ROS, which exacerbate the neuroinflammatory response (Figure 3) [88,89,90,91].

## 3. Currently FDA-Approved Therapeutic Drugs

The treatment of AD has been researched and designed for a long time, but because the cause of AD is very complex and several factors can affect the progression of the disease, current treatment for AD is still unclear [93]. There are only eight drugs approved by the FDA today for the treatment of AD, and these are not only scarce, but most of them only relieve patients’ symptoms and do not provide a therapeutic effect nor do they stop the progression of the disease. These drugs are classified into three categories according to their mechanism of action: acetylcholinesterase inhibitors (AChEIs), N-methyl-D-aspartate (NMDA) receptor antagonists, and β-amyloid-targeting drugs, which have emerged in recent years [94,95,96]. Table 1 and Table 2 summarize the basic information, target of action, mechanism of action, and drug characteristics of each drug.

### 3.1. Acetylcholinesterase Inhibitors (AChEIs)

The mechanism of action of the AChEI class of drugs is based on the hypothesis of cholinergic impairment in AD pathology. AChEIs increase acetylcholine levels and promote cholinergic neurotransmission by inhibiting acetylcholinesterase activity, thus acting as a therapeutic agent to improve cognitive dysfunction in AD patients. AChEIs are well tolerated, with mild and transient adverse effects, and are mainly used in the early or intermediate stages of AD [123,124,125].

**Figure 4:**
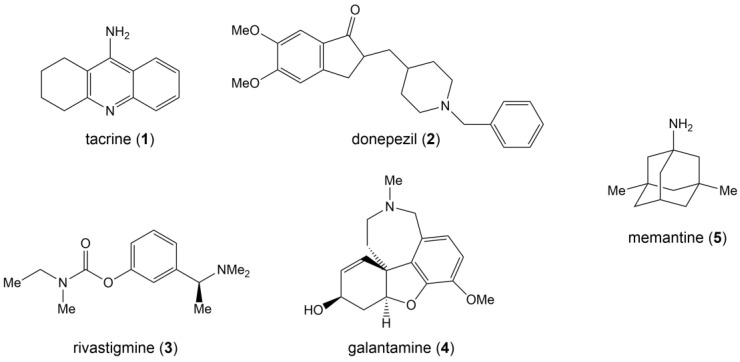
Structural formulas for five FDA-approved drugs.

The earliest FDA-approved AD treatment, tacrine (**1**), is also an AChEI but is now discontinued due to its high hepatotoxicity and short half-life [126]. AChEIs currently approved by the FDA for application include donepezil (**2**), rivastigmine (**3**), and galantamine (**4**) (Figure 4). Donepezil, also known as donepezil hydrochloride, was approved for marketing by the FDA in 1996. As a second-generation non-competitive reversible AChE inhibitor, donepezil has the advantages of high target and tissue selectivity, low adverse effects, safety and efficacy, and good tolerability, and it has been widely used in clinic settings. Rivastigmine, approved in 2000, is an inhibitor of AChE and butyrylcholinesterase (BuChE). AChE and BuChE are the two most important enzymes responsible for acetylcholine hydrolysis. In a normal brain, BuChE is primarily found in glial cells, but in an AD brain, BuChE activity rises by 40–90%, resulting in a decrease in ACh activity [127]. Rivastigmine is CNS selective and therefore has fewer side effects on the peripheral nervous system. Galantamine, approved in 2001, is a competitive reversible inhibitor and a variant ligand of nicotinic acetylcholine receptors (nAChRs) that protects neurons by binding nAChRs [104,106,109,128].

### 3.2. N-Methyl-D-aspartate (NMDA) Receptor Antagonist

The only drug in the NMDA receptor antagonist class that has received FDA approval is memantine (**5**) (Figure 4), which was approved in 2003 and became the first drug to be used in the treatment of patients with moderate to severe AD [129].

The mechanism of action of the NMDA receptor antagonist class of drugs is based on the hypothesis of glutamate stimulant toxicity in AD pathology. When NMDA receptors excessively bind to glutamate, Ca^2+^ concentrations in neurons increase and promote excitotoxicity, ultimately leading to neuronal death [130]. As a non-competitive NMDA receptor antagonist, memantine can bind NMDA receptors and produce antagonistic effects, thereby reducing intracellular Ca^2+^ levels and the excitatory neurotoxic effects of glutamate, providing neuroprotection and symptom relief. It also avoids the negative effects caused by prolonged receptor blockade due to the low affinity of meperidine [112,131,132].

A combination of AChEIs and NMDA receptor antagonists can also be used to treat AD, and this combination treatment strategy is more advantageous than monotherapy. Namzaric, which was approved by the FDA in 2014, is a combination of donepezil and memantine for the treatment of patients with moderate to severe AD [114].

### 3.3. β-Amyloid Targeting Drugs

In 2016, Sevigny et al. [116] screened aducanumab from a pool of human memory B cells through an Aβ plaque-triggered B cell cloning approach. Aducanumab is a human monoclonal antibody (mAb) with a high affinity for Aβ proteins, and it can penetrate the BBB to bind and remove Aβ proteins and reduce the deposition of Aβ plaques in the brain, thus acting as a treatment for AD [116,118,133]. PET imaging was able to observe that aducanumab reduced Aβ plaques in the brain in a dose- and time-dependent manner. In October 2019, Biogen demonstrated significant cognitive decline relief in patients belonging to a high-dose aducanumab-treated group in the EMERGE study of the Phase III trial, and as a result, Biogen planned to submit a biologics license application in 2020 [134,135]. In June 2021, aducanumab (Aduhelm) received accelerated approval from the FDA, making it the first AD treatment approved for the removal of the Aβ protein as a mechanism of action. However, clinical evidence for aducanumab has been incomplete to date, and its efficacy has been questioned by scientists and regulatory experts, making the approval of aducanumab controversial [136,137,138].

Lecanemab is the second drug to target β-amyloid for the treatment of AD. Lecanemab is also a human monoclonal antibody with a specific affinity for β-amyloid aggregates (called protofibrils), which positively affects AD pathology and slows disease progression by neutralizing and removing these Aβ aggregates [120].

The results of the CLARITY AD study of lecanemab were presented at the Alzheimer’s Disease Clinical Trials Conference in San Francisco, CA, on 29 November 2022. This is a double-blind, placebo-controlled, parallel-group 2b clinical trial in patients with mild cognitive impairment or early AD and confirmed amyloid pathology. The study showed a dose-dependent decrease in beta-amyloid in the lecanemab-treated group, and lecanemab slowed cognitive decline by 27% [122,139,140]. On 6 January 2023, lecanemab received accelerated approval from the FDA [141], becoming the latest beta-amyloid target drug to be approved.

## 4. Periodontitis and AD

Periodontitis is a chronic inflammatory disease with multi-factorial, multi-bacterial infection. As the inflammation spreads, pockets form, and this results in the further loss of supporting tissues around the teeth, including alveolar bone and periodontal ligaments, eventually leading to tooth loss [142,143]. As periodontitis has been studied in depth, there is growing evidence that periodontitis can affect systemic health status and that it is strongly associated with AD.

### 4.1. Study of the Correlation between Periodontitis and AD

Several studies have now confirmed that periodontitis is associated with AD. In a study by Giselle et al., 60 elderly subjects were divided into AD and control groups, and the relationship between AD and oral health status was illustrated by examining the oral health status of the subjects in both groups. Compared to the controls, AD subjects had fewer natural teeth [144], and periodontitis was the main cause of tooth loss, suggesting an association between AD and oral health status and periodontitis. In a retrospective study by Chen et al., a sample of 9291 periodontitis patients and 18,672 non-periodontitis patients were selected, and both groups of subjects returned between 1996–2013 until the subjects were diagnosed with AD or died. The authors found that patients with periodontitis for 10 years also had an increased risk of AD, suggesting a strong association between chronic neuroinflammation and AD [145]. By testing serum anti-periodontal bacterial antibody levels in subjects with ultimately confirmed AD versus non-AD controls, Stein et al. found that periodontal bacterial antibody levels were already elevated in AD subjects in the years before the onset of cognitive impairment, suggesting that periodontitis may increase the risk of developing AD [146]. Ide et al. recruited 60 subjects with mild to moderate AD to test their cognitive abilities, dental health, and circulating levels of inflammatory markers. Forty-three of these subjects were followed up after 6 months and retested for inflammatory marker levels, and the authors found that periodontitis in the participants was associated with increased rates of cognitive decline and inflammatory marker levels, suggesting that periodontitis advances the disease process in AD [147]. Batty et al. recruited 11,140 patients with type II diabetes aged 55–88 years and followed them regularly over the following 5 years to determine their status of cognitive decline. They found that the greater the number of missing teeth, the greater the risk of dementia and cognitive decline. This suggests that tooth loss is associated with an increased risk of cognitive decline [148,149]. Kaye et al. selected 597 male subjects between the ages of 28–70 years for a 32-year period with return visits every three years. The results showed that the risk of cognitive decline was higher for subjects aged over 45.5 years and in those with a greater number of tooth loss. This suggests that cognitive decline is associated with periodontal and oral health [150,151].

Sufficient evidence for an association between *Porphyromonas gingivalis* and AD has been provided in a study by Dominy et al. *Porphyromonas gingivalis*, the main causative agent of periodontitis, produces a virulence factor, gingipains, a cysteine protease consisting of lysine-gingipain (Kgp), arginine-gingipain A (RgpA) and arginine-gingipain B (RgpB), which can hydrolyze the Tau protein and exacerbate the hyperphosphorylation of the Tau protein. The levels of Kgp and Rgp in the brains of AD patients were significantly higher than those of non-AD patients, and the specific gene *hmuY* of *Porphyromonas gingivalis* was detected in the brains and cerebrospinal fluid of AD patients, confirming the presence of *Porphyromonas gingivalis* infection in the brains of AD patients and revealing that *Porphyromonas gingivalis* is a potential pathogenic factor for the inducing of AD [152].

Díaz-Zúñiga et al. orally injected rats with *Porphyromonas gingivalis*; assessed hippocampus-dependent spatial memory by means of the Oasis maze; and collected maxilla, cerebrospinal fluid, and hippocampus samples for evaluation from the rats that completed the maze. They found that rats with an oral infection of *Porphyromonas gingivalis* had worse spatial memory; significantly more alveolar bone loss; significantly higher levels of proinflammatory factors in cerebrospinal fluid, serum, and hippocampus; and more P-Tau staining of the CA1 hippocampal regions. Via q-PCR quantification, they identified the presence of RgpA and Kgp gingipain genes in the rat hippocampus [127,153]. Kantarci et al. investigated the status of alveolar bone loss and changes in neuroinflammatory responses in AD model mice and wild-type (WT) mice by placing silk ligatures at the maxillary second molar in mice to cause oral bacterial colonization and induce periodontitis. They found that experimental periodontitis increased alveolar bone loss in WT and 5xFAD mice and led to significantly higher levels of insoluble Aβ42 in the brains of 5xFAD mice. Experimental periodontitis also increased neuroinflammation in WT mice and triggered abnormal inflammatory regulation in the brains of 5xFAD mice [154]. Ting et al. administered Porphyromonas gingivalis outer membrane vesicles (Pg-OMVs) via gavage to mice and found that Pg-OMVs impaired memory and learning; decreased the expression of tight junction-related genes and proteins in the hippocampus; and triggered pathological features of AD such as memory dysfunction, neuroinflammation, and the phosphorylation of the Tau protein [155]. A study by Jiang et al. found that chronic systemic exposure to Porphyromonas gingivalis lipopolysaccharide advances AD disease progression, including deficits in learning and memory function, microglia-mediated neuroinflammation, and hyperphosphorylation of Tau proteins in APP^NL-F/NL-F^ mice [156].

All of these studies indicate that periodontitis is one of the risk factors for the development of AD, that *Porphyromonas gingivalis* is the main pathogen linking the two diseases, and that neuroinflammatory mechanisms are an important linking mechanism between the two diseases.

At the same time, the link between periodontitis and AD is bidirectional. While periodontitis affects the progression of AD disease, it is difficult for AD patients to maintain their own oral cleanliness and to take the initiative to receive professional dental treatment and care due to the loss of their self-care abilities. Caregivers conducting studies in hospitals have observed that AD patients may refuse to brush their teeth or forget to brush their teeth. These factors may increase the risk of periodontal bacterial infection, which can eventually trigger periodontitis and lead to tooth loss [157,158]. In a study by Aragón et al., a sample of 70 subjects with AD and 36 control subjects were selected, and an evaluation was conducted on their oral health indices, DMFT/DMFS, CPI, prosthetic conditions, oral hygiene, salivary volume, and pH, as well as specific microbiological parameters controlling the risk of dental caries. The results showed that AD subjects had poorer oral health, more mucosal lesions, and poorer saliva volume and quality [159]. D’Alessandro et al. collected dental data on dementia severity; medications; physical status; and decayed, filled, and remaining natural teeth in 120 AD subjects and 103 control subjects, and they found that the oral health of AD patients declined as the severity of the disease worsened and that gingival bleeding rates, calculus, probing depths, and gingival indices were significantly higher in the AD patients as compared to the control group [160,161]. Martande et al. assessed the periodontal health status of 58 AD subjects and 60 non-AD subjects and showed that the decrease in clinical periodontal parameters (including gingival and plaque indices, pocket depth, and bleeding rate on probing) was significantly greater in AD subjects than in non-AD subjects [162]. All of this suggests that AD has an impact on periodontal health status as well.

Table 3 summarizes descriptions of clinical studies on the association of AD with oral health and periodontitis.

### 4.2. Possible Mechanisms of Action between Periodontitis and AD

Although there is no definitive evidence today on the mechanisms by which periodontitis influences and advances the disease process of AD, the most accepted view is that periodontitis is associated with AD through a neuroinflammatory mechanism. This effect advances the disease progression of AD through both the direct invasion of periodontal pathogens into the central nervous system and through the induction of neurodegeneration by a systemic inflammatory response [163].

**Figure 5:**
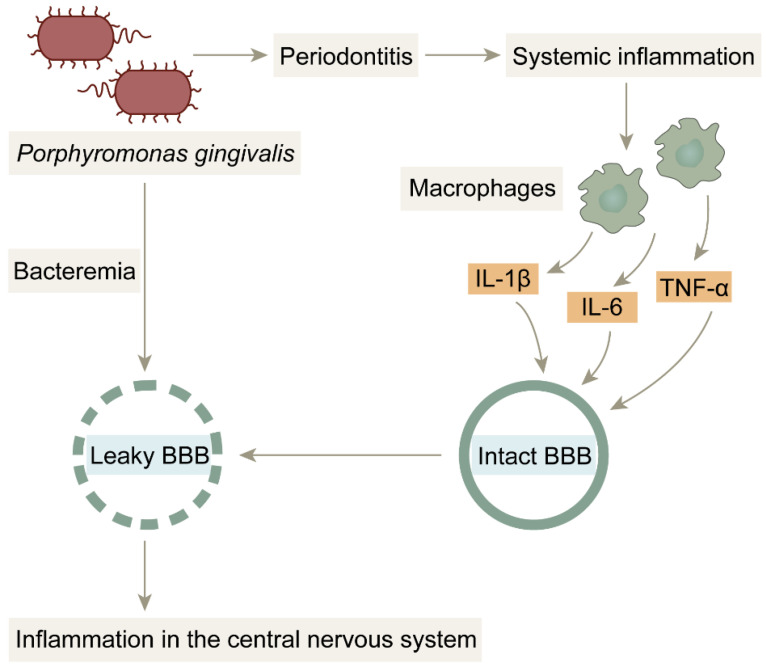
BBB damage due to bacterial infection: *Porphyromonas gingivalis* penetrates the blood–brain barrier into brain tissue by triggering bacteremia. Under normal conditions, the blood–brain barrier prevents harmful substances from entering the brain; however, in inflammatory states, the blood–brain barrier is damaged by pro-inflammatory factors and is no longer structurally intact, providing favorable conditions for *Porphyromonas gingivalis* and LPS to enter the brain.

**Figure 6:**
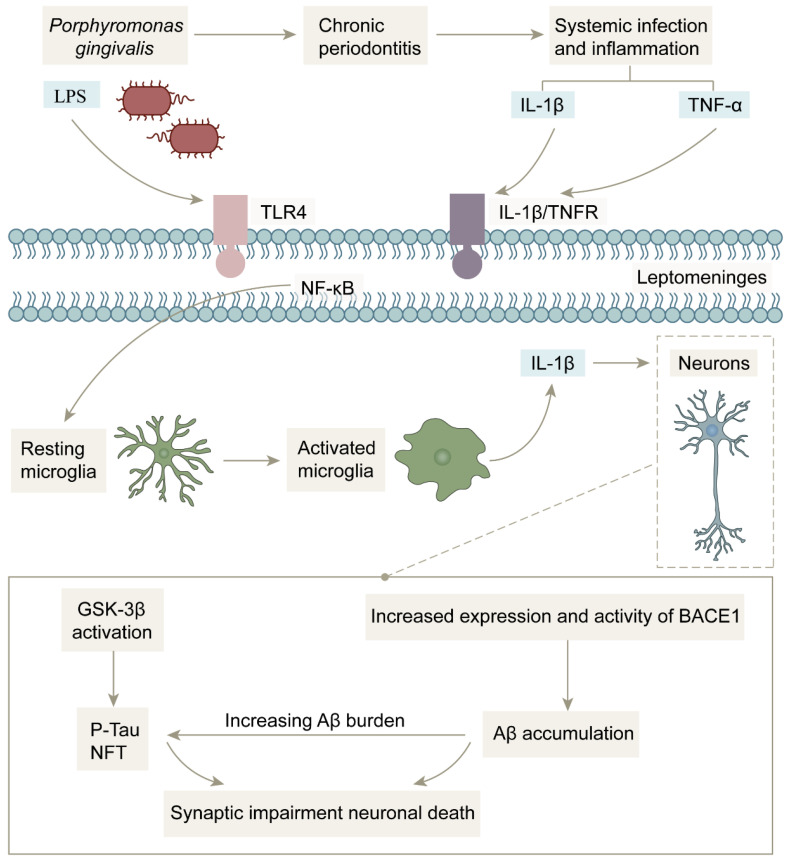
Mechanism of action of bacterial infection-induced inflammation in the central nervous system: After *Porphyromonas gingivalis* invades brain tissue, inflammatory signals and *Porphyromonas gingivalis* LPS activate microglia through leptomeninges, and activated microglia release pro-inflammatory factor IL-1β to increase BACE1 activity while activating glycogen synthase kinase 3, which exacerbates Aβ deposition and Tau protein hyperphosphorylation, ultimately leading to neuronal death.

The first is the pathway of direct invasion of periodontal pathogens into the central nervous system. The development and progression of periodontitis is associated with more than a dozen pathogenic bacteria, approximately 85% of which are Gram-negative, with *Porphyromonas gingivalis* considered to be the most critical pathogen. The periodontal barrier is disrupted under the action of periodontitis, which provides favorable conditions for the invasion of *Porphyromonas gingivalis* and its lipopolysaccharide (LPS) into the blood and nervous system [164,165,166]. *Porphyromonas gingivalis* and its LPS can also invade the bloodstream through daily actions such as brushing, chewing, flossing, or oral surgery, causing bacteremia [167] and crossing the blood–brain barrier (BBB) to the brain (Figure 5). Singhrao et al. also hypothesized that the BBB initially becomes weakened during aging, which makes it easier for Porphyromonas gingivalis to enter the brain to further damage the BBB, which in turn allows more periodontal bacteria and virulence factors to enter the CNS through the BBB [166,168], creating a vicious cycle. LPS from *Porphyromonas gingivalis* is an important factor in causing central neuroinflammation. After the invasion of the brain, LPS acts on TLR-4 receptors and CD14 on leptomeninges (Figure 6), activating NF-κB signaling and stimulating microglia to release the proinflammatory factor IL-1β. IL-1β stimulates neurons and increases BACE1 activity and expression, thereby exacerbating the deposition of Aβ. Moreover, while LPS acts on TLR-4 receptors, it also activates glycogen synthase 3, which promotes the hyperphosphorylation of the Tau protein. This series of events ultimately leads to synaptic damage and neuronal death, advancing the disease process of AD [164,165,166,169,170].

S. Poole et al. examined brain tissue from AD patients and non-AD patients by immunolabeling and immunoblotting and eventually detected the presence of *Porphyromonas gingivalis* LPS in brain tissue from AD patients 12 h after death [8]. Thus, the possibility of this pathway was also confirmed.

Kitazawa M et al. and Lee JW et al. induced chronic neuroinflammation by injecting *Porphyromonas gingivalis* LPS into an AD mouse model and found that it exacerbated the production of pro-inflammatory factors and thus promoted Aβ aggregation and Tau protein hyperphosphorylation [171,172]. In a study by Ishida et al., they induced periodontitis by inoculating a transgenic AD mouse model with *Porphyromonas gingivalis.* After comparing with control mice, they found that cognitive function was significantly impaired in mice inoculated with the bacteria, and their Aβ levels and levels of pro-inflammatory factors such as IL-1β and TNF-α were higher in the hippocampus and cortex than in control mice. In addition, they found that LPS levels in the serum and brain of the bacteria-infected mice were significantly higher than those of the control mice by measuring [10]. Ilievski et al. and Singhrao et al. found Porphyromonas gingivalis and gingipains in frontotemporal lobe and hippocampal tissue sections from mice orally infected with Porphyromonas gingivalis. In addition, Porphyromonas gingivalis and gingipains were also present in microglia, astrocytes, and neurons, suggesting that they mediate neuroinflammatory activity through glial cells and neuronal cells [166,167,168]. These studies all suggest that *Porphyromonas gingivalis* and its LPS participate in and advance the pathogenesis of AD by triggering inflammation in brain tissue.

The second is the induction of the neurodegenerative pathway through a systemic inflammatory response. Infection by periodontal pathogenic microorganisms stimulates the secretion of large amounts of pro-inflammatory cytokines, such as TNF-α, IL-1β, and C-reactive protein (CRP), which dramatically increase their concentrations in the systemic circulation and lead to a prolonged state of systemic inflammation. These peripheral pro-inflammatory factors can cross the blood–brain barrier through the systemic circulation into the central nervous system, where inflammatory signals are transmitted to microglia in the brain through the leptomeninges. Activated microglia secrete IL-1β to increase the activity of BACE1, which ultimately causes neuronal functional impairment (Figure 6). In addition, peripheral pro-inflammatory factors can also affect the brain through neural pathways, the main means of which occurs via further increasing the level of pro-inflammatory factors in brain tissue by stimulating trigeminal nerve fibers, contributing to Aβ deposition and Tau protein hyperphosphorylation, inducing neuronal degeneration, and ultimately leading to cognitive decline [5,163,173,174,175].

## 5. Conclusions and Outlook

To date, the pathogenesis of AD remains a key issue for researchers to explore. The pathogenesis of AD is extraordinarily complex, involving not only multiple signaling molecular pathways but also interconnections and interactions among the pathogenic mechanisms, and the multiple hypotheses proposed so far have failed to provide a complete and comprehensive explanation of AD pathogenesis. This complexity has also led to a lack of therapeutic tools and drugs for AD. The only two classes of drugs approved by the FDA are AChEIs and NMDA receptor antagonists, which can only improve the symptoms of cognitive dysfunction but not change the disease process.

Recent years have seen significant breakthroughs in the development and research of drugs for AD. FDA-approved aducanumab in 2021 and 2023’s newly approved lecanemab became the first drugs to target the pathophysiology of AD, acting by directly removing Aβ, a feature of AD pathology. These drugs not only treat disease symptoms but can also influence and modify AD disease progression. The approval of aducanumab and lecanemab not only offers new hope for the treatment of AD but also provides directions and ideas for new drug development: firstly, targeting Aβ and targeting phosphorylated Tau protein can be an important direction for new drug development based on the two major pathological features of AD. For example, by using GSK-3β as a drug target, the hyperphosphorylation of the Tau protein can be inhibited via inhibiting the activity of GSK-3β or reducing the formation of NFT by inhibiting the intracellular aggregation of Tau to achieve treatment of AD at a pathophysiological level. Secondly, drug targets can be identified for the currently proposed hypothesis of AD pathogenesis. Combination studies on existing drugs can also be conducted to find more effective therapeutic strategies.

Current research on the pathological mechanism of AD infection is also very popular. As research progresses, a growing amount of evidence indicates that AD is associated with periodontitis and *Porphyromonas gingivalis* infection, and this connection is of great importance and can provide new ideas for finding a treatment for AD. Periodontitis is a treatable disease compared to AD. Therefore, the risk of AD can be reduced or the progression of AD can be indirectly mitigated by raising awareness of oral protection and keeping teeth clean and healthy. Although a large amount of evidence suggests that periodontitis is involved in the advancement of AD disease progression, the mechanism of interaction between the two diseases has not yet been clarified, and it is still necessary for researchers to conduct in-depth studies and identify new and effective treatments, which are of great importance for the early diagnosis and treatment of AD and for slowing down disease progression.

## Figures and Tables

**Table 1:** Summary of basic information on drugs currently approved by the FDA [97].

Drug Name	Band Name	Category	Development Company	Time of FDA Approval
Tacrine	Cognex	Acetylcholinesterase inhibitors (AChEIs)	Warner-Lambert	1993
Donepezil	Aricept	Acetylcholinesterase inhibitors (AchEIs)	Eisai	1996
Rivastigmine	Exelon	Acetylcholinesterase inhibitors (AchEIs)	Novartis AG	2000
Galantamine	Razadyne	Acetylcholinesterase inhibitors (AchEIs)	Sopharma Trading	2001
Memantine	Namenda	N-methyl-D-aspartate (NMDA) receptor antagonist	Lundbeck	2003
Donepezil and Memantine	Namzaric	Co-medication	Actavis	2014
Aducanumab	Aduhelm	Removal of Aβ protein	Eisai and Biogen	2021
Lecanemab	Leqembi	Removal of Aβ protein	Eisai and Biogen	2023

**Table 2:** Summary of the targets, mechanisms, and characteristics of drugs currently approved by the FDA.

Drug Name	Molecular Target Receptors/Proteins /Enzymes	Applicable Treatment Period	Drug Effect	Action Mechanism	Drug Advantages and Characteristics	Adverse Effects
Tacrine	AChE	Currently discontinued	Relief of symptoms	Binds to the hydrophobic region of the active surface of acetylcholinesterase, thereby inhibiting its activity and increasing the level of ACh in the brain [98,99].	Wide range of targets and pathways [100].	With hepatotoxicity: elevated serum alanine aminotransferase (ALT) levels. Cholinergic effects: gastrointestinal reactions such as vomiting, dyspepsia, and diarrhea [101].
Donepezil	AChE	Mild to moderate AD	Relief of symptoms	Inhibits acetylcholinesterase activity for the purposes of alleviating neuronal degeneration caused by cholinergic impairment. Upregulating nicotinic receptors in the cortex to reduce glutamatergic neurotoxicity. Affecting APP processing to reduce Aβ neurotoxicity [102,103].	High selectivity for targets and tissues, low adverse effects, long half-life, safe and effective, and well tolerated [104].	Nausea, vomiting, and diarrhea can occur at high doses [105].
Rivastigmine	AChE BuChE	Mild to moderate AD	Relief of symptoms	Inhibits both acetylcholinesterase and butyrylcholinesterase for up to 10 h and increases the brain levels of ACh and BuChE [106,107].	Has central nervous system selectivity, with fewer peripheral side effects. Dual inhibitors of AChE and BuChE, and has advantages over AChE inhibitors [106,108].	Cholinergic effects: gastrointestinal reactions such as nausea, vomiting, diarrhea, and anorexia [108].
Galantamine	AchE nAChRs	Mild to moderate AD	Relief of symptoms	Inhibits acetylcholinesterase and increases acetylcholine concentration. Stimulates nicotinic receptors to release more acetylcholine in the brain [109].	Competitive inhibitor, variant ligand of nicotinic receptors. Helps protect neurons and enhance neurotransmitter release [109].	Cholinergic effects: gastrointestinal reactions such as nausea, vomiting, and diarrhea.
Memantine	NMDAR	Moderate to severe AD	Relief of symptoms	Binds to NMDA receptors and exerts antagonistic effects, reducing intracellular Ca^2+^ levels and the excitatory neurotoxicity of glutamate [110,111].	Safe and effective, well tolerated, with low affinity, avoiding negative learning and memory-related effects due to prolonged receptor blockade [112].	Bradycardia, weakness, convulsions [113].
Donepezil and Memantine	AChE NMDAR	Moderate to severe AD	Relief of symptoms	Increases ACh levels. Reduces glutamate excitatory neurotoxicity.	Combination of drugs has advantages over single-drug therapy.	Adverse reactions associated with donepezil and memantine [114].
Aducanumab	Soluble oligomers and insoluble proto-fibers in Aβ	Early AD	Changing the course of disease development	Penetrates the BBB, selectively binds to and removes Aβ protein from soluble oligomers and insoluble protofibrils in Aβ aggregates; reduces Aβ plaque deposition in the brain [115,116].	High affinity for Aβ protein; the first drug to target β-amyloid in the brain [117,118].	ARIA edema, microhemorrhage, headache, dizziness, nausea, diarrhea, hypersensitivity reactions, etc. [118,119].
Lecanemab	Soluble Aβ aggregates	MCI, Early AD	Changing the course of disease development	Binds to soluble Aβ aggregates; neutralizes and promotes the clearance of Aβ aggregates [120].	Well tolerated [121].	ARIA edema, microhemorrhage [122].

**Table 3:** Summary of clinical trials on the association of AD with periodontitis.

Clinical Trial Models	Trial Methods	Results	Conclusions	Authors	Reference
60 elderly subjects, divided into control and AD groups.	Testing the oral health status of subjects in both groups.	AD subjects had fewer natural teeth.	AD associated with oral health status.	Giselle et al.	[144]
9291 patients with periodontitis and 18,672 patients without periodontitis.	Two groups of subjects were returned between 1996 and 2013.	Patients who had had periodontitis for 10 years or more were also at increased risk for AD.	There is a link between chronic neuroinflammation and AD.	Chen et al.	[145]
Subjects with final diagnosis of AD patients and control subjects.	Testing the serum anti-periodontal bacterial antibody levels in both groups of subjects.	AD subjects had elevated levels of periodontal bacterial antibodies several years before the onset of cognitive impairment.	Periodontitis increases the risk of AD.	Stein et al.	[146]
60 subjects with mild and moderate AD.	Testing subjects for cognitive ability, dental health, and inflammatory marker levels.	Cognitive decline and elevated levels of inflammatory markers were associated with periodontitis in the subjects.	Periodontitis advances the disease process in AD.	Ide et al.	[147]
11,140 type II diabetic patients aged 55–88 years.	Regular follow-up over the next 5 years.	The greater the number of missing teeth a patient had, the greater the risk of dementia and cognitive decline.	Tooth loss is associated with an increased risk of cognitive decline.	Batty et al.	[148]
597 male subjects between 28 and 70 years of age.	Return visits every 3 years for a period of 32 years.	The risk of cognitive decline was higher for subjects aged over 45.5 years and the number of teeth lost increased.	Cognitive decline is associated with dental health status.	Kaye et al.	[150]
AD subjects and non-AD subjects.	Testing the brain and cerebrospinal fluid in subjects.	The levels of Kgp and Rgp in the brains of AD subjects were significantly higher than those of non-AD subjects, and the specific gene *hmuY* of *Porphyromonas gingivalis* was detected in the brains and cerebrospinal fluid of AD patients.	Porphyromonas gingivalis is a potential causative factor in the predisposition to AD.	Dominy et al.	[152]
70 AD subjects and 36 control subjects.	Evaluating oral health indices, DMFT/DMFS, CPI, prosthetic conditions, oral hygiene, saliva volume and pH, and specific microbiological parameters for control caries risk assessments.	AD subjects had poorer oral health, more mucosal lesions, and poorer saliva quantity and quality.	AD has an impact on periodontal and oral health.	Aragón et al.	[159]
120 AD subjects and 103 control subjects.	Collecting data on subjects’ dementia severity; medications; physical status; and decayed, filled, and remaining natural teeth.	The oral health of AD patients declined as the severity of the disease worsened, and gingival bleeding rates, calculus, probing depths, and gingival indices were significantly higher in the AD patients as compared to the control group.	AD has an impact on periodontal and oral health.	D’Alessandro et al.	[160,161]
58 AD subjects and 60 control subjects.	Evaluating the subjects’ clinical periodontal parameters.	The decrease in clinical periodontal parameters was significantly greater in AD subjects than in non-AD subjects.	AD has an impact on periodontal and oral health.	Martande et al.	[162]
